# Structure-Guided Functional Annotation of the Influenza A Virus NS1 Protein Reveals Dynamic Evolution of the p85β-Binding Site during Circulation in Humans

**DOI:** 10.1128/JVI.01081-17

**Published:** 2017-10-13

**Authors:** Antonio M. Lopes, Patricia Domingues, Roland Zell, Benjamin G. Hale

**Affiliations:** a Institute of Medical Virology, University of Zurich, Zurich, Switzerland; b Institute of Medical Microbiology, Jena University Hospital, Jena, Germany; St. Jude Children's Research Hospital

**Keywords:** Akt signaling, NS1, evolution, influenza virus, protein structure-function, virulence

## Abstract

Rational characterization of virulence and host-adaptive markers in the multifunctional influenza A virus NS1 protein is hindered by a lack of comprehensive knowledge about NS1-host protein protein interfaces. Here, we surveyed the impact of amino acid variation in NS1 at its structurally defined binding site for host p85β, a regulator of phosphoinositide 3-kinase (PI3K) signaling. Structure-guided alanine scanning of all viral residues at this interface defined 10 positions contributing to the interaction, with residues 89, 95, 98, 133, 145, and 162 being the most important. A bioinformatic study of >24,000 publicly available NS1 sequences derived from viruses infecting different hosts highlighted several prevalent amino acid variants at the p85β interface that either enhanced (I95) or weakened (N135, T145, L161, Y161, S164) p85β binding. Interestingly, analysis of viruses circulating in humans since the 1918 pandemic revealed the temporal acquisition of functionally relevant variants at this interface. I95 (which enhanced p85β binding) quickly became prevalent in the 1940s and experimentally conferred a fitness advantage to a recombinant 1930s-based H1N1 virus in human lung epithelial cells. Surprisingly, H1N1 and H3N2 viruses recently acquired T145 or N135, respectively, which diminished p85β binding but apparently not the overall fitness in the human population. Evolutionary analyses revealed covariation of the NS1-p85β binding phenotype in humans with functional changes at multiple residues in other viral proteins, suggesting an unexplored compensatory or synergistic interplay between phenotypes *in vivo*. Overall, our data provide a resource to understand the consequences of the NS1-p85β binding spectrum of different influenza viruses and highlight the dynamic evolution of this property in viruses circulating in humans.

**IMPORTANCE** In humans, influenza A viruses are responsible for causing seasonal epidemics and occasional pandemics. These viruses also circulate and evolve in other animal species, creating a reservoir from which novel viruses with distinct properties can emerge. The viral nonstructural protein NS1 is an important host range determinant and virulence factor that exhibits strain-specific interactions with several host factors, although few have been characterized extensively. In the study described here, we comprehensively surveyed the impact of natural and unnatural NS1 variations on the binding of NS1 to host p85β, a subunit of phosphoinositide 3-kinase that regulates intracellular metabolism and contributes to virus replication and virulence. We define the p85β-binding site on NS1 and provide a predictive resource to assess this ability of NS1 in viruses from different hosts. Strikingly, we uncover a spectrum of p85β binding by different NS1 proteins and reveal that viruses evolving in humans have undergone dynamic changes in this NS1 function over the last century.

## INTRODUCTION

The influenza A virus (IAV) NS1 protein plays a critical role in reprogramming host cell signaling pathways during infection to promote efficient viral replication (reviewed in references 1 to 3). A major function of NS1 is to directly antagonize host interferon (IFN) responses by preventing the full activation of RIG-I, either by sequestering away its double-stranded RNA trigger or by interfering with regulatory posttranslational modifications mediated by the E3 ubiquitin ligases TRIM25 and Riplet (4–7). In addition, some NS1 proteins can indirectly prevent IFN synthesis by inhibiting almost all cellular gene expression in infected cells, a property related to binding the host pre-mRNA processing machinery component CPSF30 (8, 9). NS1 proteins have also been reported to engage with several other factors to manipulate the host environment, including the p85β regulatory subunit of phosphoinositide 3-kinase (PI3K) (10–13), PAF1 (14), DDX21 (15), PACT (16), protein kinase R (17), Crk/CrkL (18, 19), and several PDZ-domain containing proteins (20–23).

Variation in the NS1 sequence has been associated with host range restriction and virulence (24), and an emerging concept is that NS1 proteins can have distinct functional properties depending upon the particular viral strain or host species of isolation. A prime example of this is the interaction of NS1 proteins with host PDZ domain-containing proteins, which occurs via a specific 4-amino-acid motif at the C terminus of NS1 that is predominantly found in avian influenza A viruses (23). A functional PDZ domain-binding motif in NS1 can enhance virulence (25), and the short, linear nature of this well-characterized interaction motif means that certain predictions regarding the pathobiology of newly emerging influenza A viruses can be made (26, 27). In contrast, the strain-specific interaction of NS1 with host CPSF30 can be more difficult to predict in emerging viruses, as the binding interface is much larger and nonlinear (28). Experimental studies have shown that the genomes of many human-adapted influenza A viruses encode NS1 proteins able to bind and inhibit the normal function of CPSF30 (29); however, prominent strains that lack this property have been able to cross the species barrier and infect humans, including the avian H5N1 virus (30), avian H7N9 virus (31), and swine-origin 2009 pandemic H1N1 virus (32, 33). In addition, H3N2 influenza A viruses expressing NS1 variants with destabilized CPSF30 binding have recently been identified in human patients (34, 35). In many virus backgrounds, CPSF30 binding contributes to virulence (31, 35, 36), and although the specific NS1 amino acid changes that diminish CPSF30 binding differ greatly between strains, the availability of a crystal structure (28) and multiple NS1-CPSF30 binding studies (29–33, 35, 37, 38) have created large reference data sets to aid in evaluating the pathogenic potential of newly identified influenza A viruses on the basis of their NS1 sequences.

Other than CPSF30, the only known host protein with a structurally defined interface with NS1 is the p85β regulatory subunit of PI3K (39). Binding of NS1 to p85β activates the PI3K signaling pathway in infected cells (10), and artificial mutations in NS1 that destabilize this interaction attenuate virus replication *in vitro* and virulence *in vivo* (10, 40–42). A single naturally occurring amino acid variant in the NS1 proteins of some highly pathogenic H5N1 avian influenza virus strains has been associated with alteration of the interaction of NS1 with p85β and an impact on virulence under certain circumstances (43); however, the existence and prevalence of other functionally similar variants are unknown. The biological consequences that occur downstream of NS1-activated PI3K are also not fully understood: triggering of cation conductance in the infected lung epithelium has been suggested (44), and suppression of virus-induced apoptosis has been controversial (11, 41, 45), possibly due to the use of different virus strains and a lack of knowledge surrounding how different NS1 variants from these strains engage with PI3K. In this study, we sought to comprehensively define the p85β-binding site in NS1, creating a resource of loss-of-function mutants for further phenotypic study. We also sought to assess the impact of natural NS1 variants on this activity, both to investigate potential host-adaptive strategies and to create a framework for the future rapid characterization of newly emerging influenza A viruses.

## RESULTS

### Identification of NS1 residues that contribute to p85β binding.

**FIG 1:**
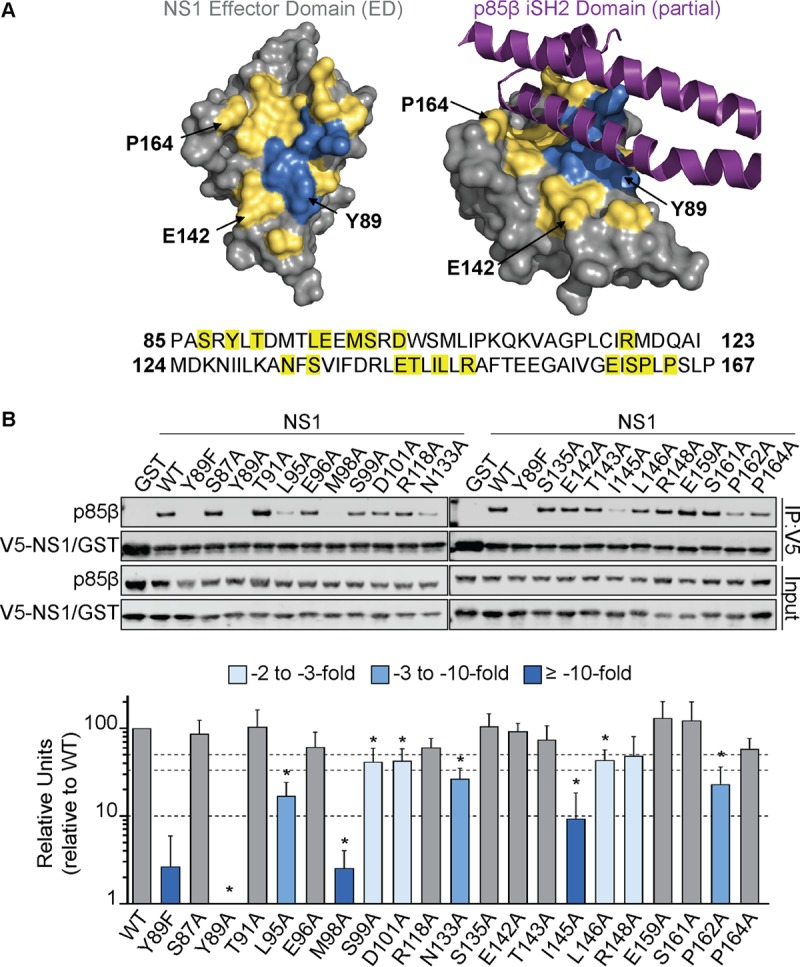
Identification of NS1 residues that contribute to p85β binding. (A) Crystal structure of the NS1 effector domain (ED; silver) alone (left) or in complex with a portion of the p85β iSH2 domain (purple) (right). The 20 NS1 residues that contact p85β are indicated in yellow (see also the NS1 sequence at the bottom of panel A). Three NS1 residues previously suggested to be important for this interaction are labeled, while the locations of the four most critical residues identified in this study (Y89, L95, M98, and I145) are in blue. The figure was generated using the PyMOL program (PDB accession number 3L4Q). (B) (Top) Western blot analysis of anti-V5 immunoprecipitates (IP) from lysates of 293T cells expressing V5-GST or various V5-PR8 NS1 single amino acid mutants together with FLAG-p85β. Proteins of interest were detected using specific antibodies. Input samples were similarly processed. (Bottom) Band intensity was quantified using a Li-Cor Odyssey scanner. The mean numbers of relative units from three independent experiments are plotted. Error bars represent standard deviations (SDs). Significance was determined by Students *t* test (*, *P* < 0.00385). WT, wild type.

Analysis of the previously published crystal structure of the NS1 effector domain in complex with its primary binding site on p85β revealed that 20 NS1 residues directly contact p85β (39) (Fig. 1A). Of these, 3 residues (Y89, E142, and P164) have been previously implicated in p85β binding (10, 41, 46), although E142 and P164 have only ever been tested for their contribution in combination with other residues not found at the interface (13, 41, 46). Notably, F138, the NS1 residue linked to modulation of the p85β interaction in some H5N1 viruses (43), is not a direct interface residue, suggesting more complex mechanisms by which changes at this position alter virulence. We generated a library of V5-tagged A/Puerto Rico/8/1934 (H1N1) (PR8) NS1 constructs with individual alanine substitutions at each of the 20 residues that contact p85β and tested their abilities to bind FLAG-tagged human p85β in coimmunoprecipitation assays following cotransfection in 293T cells. As previously described (10), wild-type V5-tagged NS1 efficiently precipitated p85β, unlike either the NS1 Y89F mutant or a glutathione *S*-transferase (GST) control (Fig. 1B). Of the 20 PR8 NS1 single alanine mutants tested, we observed that 10 exhibited a loss-of-function phenotype with respect to p85β binding (arbitrarily defined as a ≥2-fold reduction in the level of p85β binding compared to that of the wild type): Y89A, L95A, M98A, S99A, D101A, N133A, I145A, L146A, R148A, and P162A (Fig. 1B). These loss-of-function mutants had a spectrum of p85β binding: compared to the wild type, the S99A, D101A, L146A, and R148A mutants showed 2- to 3-fold reductions in binding, while the L95A, N133A, and P162A mutants showed 3- to 10-fold reductions. The I145A mutant exhibited a >10-fold reduction in binding, while the Y89A and M98A mutations had the greatest impact (>100-fold and >30-fold reductions, respectively) (Fig. 1B). The M98A mutant had a p85β-binding capacity similar to that of the well-characterized NS1 Y89F mutant, but the NS1 Y89A mutation abrogated the p85β-binding capacity to an even greater extent (Fig. 1B). Strikingly, of the 3 previously reported contact residues, only Y89 was found to be important for p85β binding: single alanine substitutions at position 142 or 164 had a minimal impact in our experiments and did not reach the arbitrary 2-fold threshold. These data rationally map 10 PR8 NS1 residues that contribute to p85β binding, and 9 of these are newly described. Notably, the most important contact residues (Y89, L95, M98, and I145) cluster together in the three-dimensional structure of NS1, forming a hydrophobic groove into which the inter-SH2 (iSH2) domain of p85β is inserted (Fig. 1A).

### Impact of naturally occurring NS1 variants on p85β binding.

**FIG 2:**
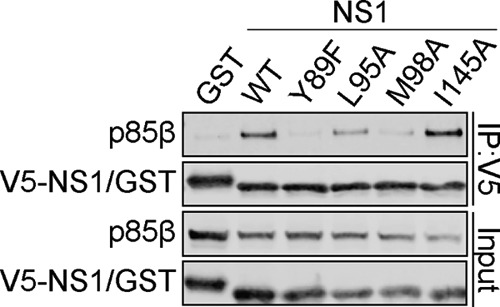
Assessment of 2009 pandemic H1N1 NS1 residues that contribute to p85β binding. The results of a Western blot analysis of anti-V5 immunoprecipitates from lysates of 293T cells expressing V5-GST or various V5-Cal09 NS1 single amino acid mutants together with FLAG-p85β are shown. Proteins of interest were detected using specific antibodies. Input samples were similarly processed. Data are representative of those from three independent experiments.

**FIG 3:**
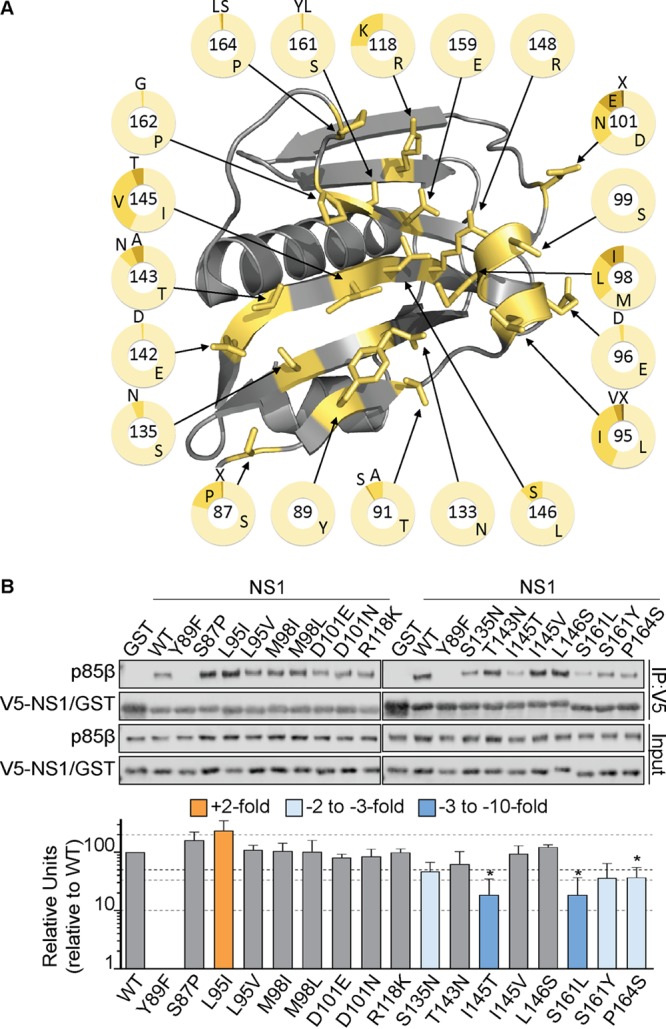
Impact of naturally occurring NS1 variants on p85β binding. (A) Crystal structure of the NS1 effector domain (silver) highlighting naturally occurring variants among the 20 p85β contact residues (PDB accession number 3L4Q). Each residue is labeled, and the pie charts indicate the prevalence of various amino acids at each position among ∼24,000 IAV NS1 sequences obtained from the NIAID Influenza Research Database. (B) (Top) Western blot analysis of anti-V5 immunoprecipitates from lysates of 293T cells expressing V5-GST or various V5-NS1 single amino acid mutants together with FLAG-p85β. Proteins of interest were detected using specific antibodies. Input samples were similarly processed. Band intensity was quantified using a Li-Cor Odyssey scanner. (Bottom) The mean numbers of relative units from three independent experiments are plotted. Error bars represent standard deviations (SDs). Statistical significance was determined by the Student *t* test (*, *P* < 0.004545).

The critical contributions of Y89, L95, and M98 (but, interestingly, not the contribution of I145) to p85β binding were confirmed for the 2009 pandemic H1N1 virus NS1 protein (Fig. 2), suggesting both common and unique features of different NS1 proteins with respect to this phenotype. Using >24,000 publicly available sequences deposited to the NIAID Influenza Research Database (IRD) (47), we therefore further investigated the naturally occurring variation in NS1 proteins from diverse influenza A viruses, with a focus on the 20 amino acid residues that make up the p85β-binding site. To minimize sampling bias, we excluded the large number of 2009 pandemic H1N1-derived virus sequences and considered these viruses separately. As graphically summarized in Fig. 3A, some of the NS1 residues at this interface are extremely well conserved, including S99, N133, E142, R148, E159, and P162, as well as the critical Y89 residue. In contrast, we observed variations at the three other most important p85β contact residues in NS1: 95, 98, and 145. Variants at these positions (e.g., 95L/I/V, 98M/L/I, and 145I/V) largely maintained hydrophobicity, although notably, 6% of NS1 proteins had 145T, potentially disrupting the nature of the p85β-binding site. Several other sites exhibited variations that resulted in considerable physicochemical differences, such as 87S/P, 101D/E/N, 118R/K, 135S/N, 143T/N/A, 146S/L, 161S/Y/L, and 164P/L/S. We generated V5-tagged PR8 NS1 constructs with each individual variant observed at the 20 p85β contact residues, focusing on those variants with a high prevalence (>1%), apparent host species specificity, or distinct side chain properties. The abilities of these mutants to bind FLAG-tagged human p85β in coimmunoprecipitation assays following cotransfection into 293T cells were then assessed. No single naturally occurring amino acid variant tested completely abrogated the binding of NS1 to p85β (Fig. 3B). However, 5 NS1 variants appeared to destabilize (by ∼2- to 5-fold) the p85β interaction: 135N, 145T, 161L/Y, and 164S (Fig. 3B). Interestingly, a single amino acid variant (95I) consistently resulted in enhanced p85β binding (>2-fold) (Fig. 3B). These data largely complement and confirm our alanine scan-derived observations that a hydrophobic patch consisting of residues 89, 95, 98, and 145 constitutes a critical core of the p85β-binding site. This patch is highly conserved, maintaining hydrophobic properties, and natural and unnatural variants that negate hydrophobicity serve to disrupt p85β binding. Furthermore, our variant analysis revealed an important role for residues 135, 161, and 164 that was not apparent from the alanine scan data alone.

### NS1 p85β-binding variants have arisen in human IAVs and have functional consequences.

**FIG 4:**
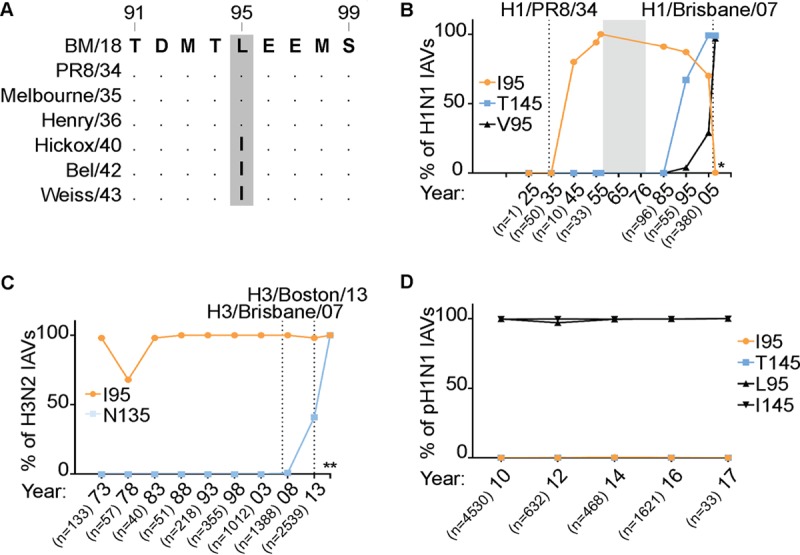
NS1 p85β-binding variants have arisen in human IAVs. (A) Sequence alignment of NS1 proteins from early human H1N1 IAVs highlighting the emergence of 95I. BM18, A/Brevig Mission/1/1918 (H1N1); Melbourne/35, A/Melbourne/1935 (H1N1); Henry/36, A/Henry/1936 (H1N1); Hickox/40, A/Hickox/1940 (H1N1); Bel/42, A/Bel/1942 (H1N1); Weiss/43, A/Weiss/1943 (H1N1). (B) Graph indicating the evolution of residues 95 and 145 in H1N1 IAVs (excluding the 2009 pandemic virus). H1/Brisbane/07, A/Brisbane/59/2007 (H1N1). The percentage of isolates harboring a particular residue within the indicated period is plotted. n, the number of isolates analyzed per time point; *, results for the years 2006 to 2008 (data are for 708 isolates). Selected strains are highlighted. (C and D) Same as panel B, but for H3N2 (C) and 2009 pandemic H1N1 (D) viruses. H3/Boston/13, A/Boston/DOA2-172/2013 (H3N2); H3/Brisbane/07, A/Brisbane/10/2007 (H3N2). **, results for the years 2014 and later (data are for 2,200 isolates).

**FIG 5:**
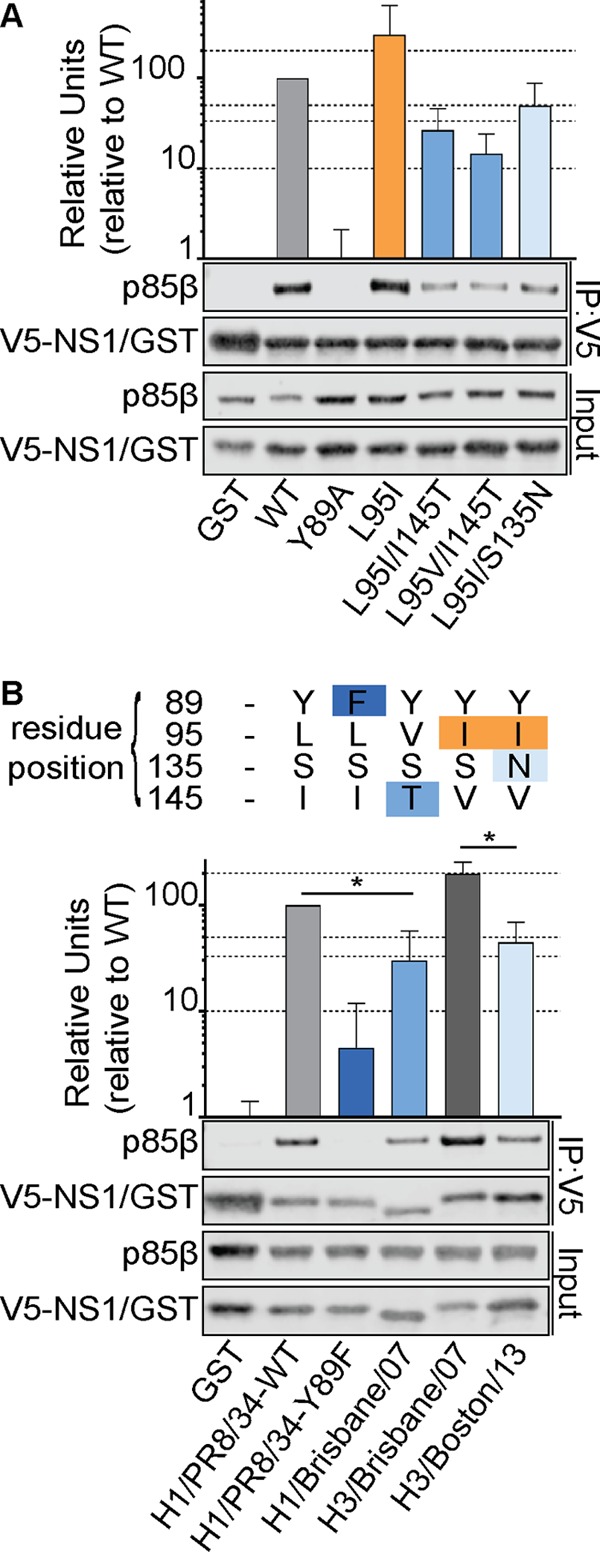
Human IAV NS1 p85β-binding variants have functional consequences. (A) Western blot analysis of anti-V5 immunoprecipitates from lysates of 293T cells expressing V5-GST or various V5-NS1 mutants together with FLAG-p85β. (B) Same as panel A, but including V5-NS1 proteins derived from the indicated virus strains. The sequences at key residues are indicated at the top. For both panel A and panel B, proteins of interest were detected using specific antibodies. Input samples were similarly processed. Band intensity was quantified using a Li-Cor Odyssey scanner. The mean numbers of relative units from three independent experiments are plotted. Error bars represent standard deviations (SDs). Significance was determined by the Student *t* test (*, *P* < 0.05).

We focused on variants at positions 95, 135, and 145 that promoted or destabilized p85β binding, as they were commonly found in either previously or currently circulating human IAVs. Further analysis of the prevalence of these variants revealed that 95I, which can enhance p85β binding, rapidly arose in NS1 proteins derived from circulating human H1N1 IAVs in the 1940s (Fig. 4A and B) and was present in the majority of human H1N1 viruses for several decades until the 2000s, after which it was gradually replaced by 95V, which is a weaker p85β-binding residue than 95I (Fig. 4B). As a consequence of 95I appearing in H1N1 viruses in the 1940s, this variant is also found in the descendant NS1 proteins of essentially all human H3N2 IAVs, where it has been maintained (Fig. 4C). Interestingly, in parallel with the disappearance of 95I, 145T (a residue that weakens p85β binding) emerged in the NS1 proteins of seasonal human H1N1 viruses, while 135N, another residue in NS1 that weakens p85β binding, has become dominant in human H3N2 viruses since 2010. To date, 2009 pandemic-derived seasonal H1N1 viruses have maintained their 1918-like classical swine H1N1 lineage NS1 sequence at these sites (Fig. 4D). We tested the impact of these residues that weaken p85β binding, 145T and 135N, on the ability of the NS1 95I construct to interact with p85β, thus mimicking the natural evolution of the p85β-binding site in NS1 in human IAVs. We observed that both of these variants independently had a negative effect on NS1 binding to p85β in the context of 95I, indicating that the residues that weaken p85β binding are dominant (Fig. 5A).

From our data, we would predict that NS1 proteins from H1N1 IAVs isolated in the late 2000s would have a weaker p85β-binding capacity than NS1 proteins from early H1N1 viruses (i.e., PR8, the reference strain used in this study) because of the presence of the 95V and 145T variant residues. In contrast, we would expect that NS1 proteins from H3N2 viruses isolated at about the same time would have a much higher p85β-binding capacity due to the presence of 95I, but NS1 proteins from later H3N2 viruses would have diminished binding due to the appearance of 135N. We tested this by performing p85β coimmunoprecipitation experiments with V5-tagged NS1 proteins derived from human strains A/Brisbane/59/2007 (H1N1), A/Brisbane/10/2007 (H3N2), and A/Boston/DOA2-172/2013 (H3N2) (Fig. 5B). In agreement with our predictions, the pattern of binding of these NS1 proteins to p85β recapitulated the effects seen with single and double variant mutations in our reference NS1 construct. These data indicate that NS1 variation at allosteric sites that may also have accumulated over time has no compensatory impact on the p85β-binding capacity, and suggest that IAV NS1 proteins that have evolved in humans over the last century exhibit temporal variation in their p85β-binding capacity that is predictable on the basis of our new functional annotation of the p85β-binding site.

### NS1 p85β-binding variants in human IAVs do not switch p85 isoform specificity.

**FIG 6:**
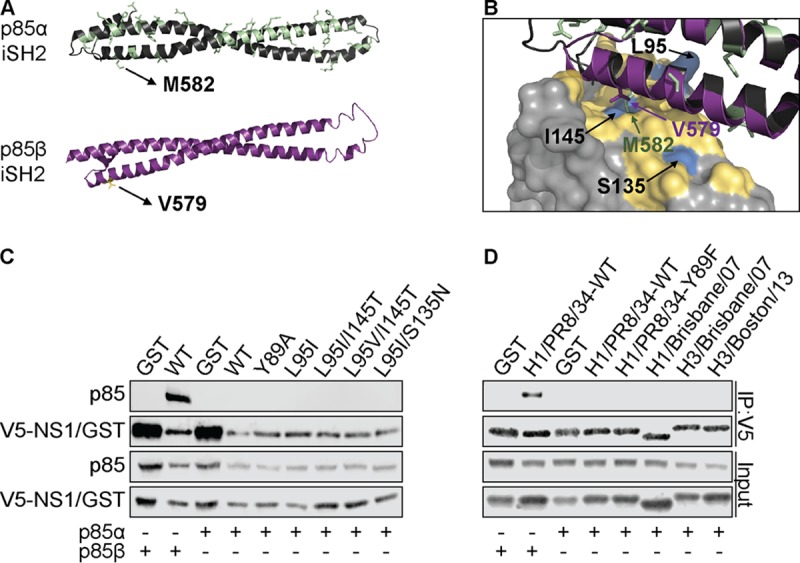
Human IAV NS1 p85β-binding variants do not switch p85 isoform specificity. (A) Structures of the iSH2 domains of p85α (green; PDB accession number 2V1Y) and p85β (purple; PDB accession number 3L4Q). Residues of the p85α iSH2 domain that differ from the p85β sequence are highlighted. Human M582 (p85α) and V579 (p85β) are labeled as potential contact residues with NS1. (B) Close-up of the interface between NS1 and p85β (PDB accession number 3L4Q). Critical NS1 residues with functional variants are labeled, and the p85α iSH2 domain is superpositioned to indicate the relative positioning of p85α M582. (C) Western blot analysis of anti-V5 immunoprecipitates from lysates of 293T cells expressing V5-GST or various V5-NS1 mutants together with either FLAG-p85α or FLAG-p85β. (D) Same as panel C, but including V5-NS1 constructs derived from the indicated strains. For both panel C and panel D, proteins of interest were detected using specific antibodies. Input samples were similarly processed. Data are representative of those from at least two independent experiments.

We considered that the p85β-binding variation in NS1 could be a result of adaptive mutations allowing NS1 to bind another human host protein. p85α is an alternative regulatory subunit of class IA PI3Ks, and its iSH2 domain is highly homologous to that of p85β, although strikingly, this subunit is not targeted by NS1 (10). Indeed, it has previously been shown that only a single amino acid change in this domain of p85α (M582V) can allow the binding of NS1 (Fig. 6A) (46). Notably, NS1 residue 145 lies in close proximity to this binding determinant (Fig. 6B); therefore, we tested whether any of the human IAV NS1 variants had gained the capacity to bind p85α. In coimmunoprecipitation assays, wild-type V5-tagged NS1, but not a GST control, was able to precipitate FLAG-tagged p85β, but neither V5-tagged construct could precipitate FLAG-tagged p85α (Fig. 6C). Similarly, isogenic V5-tagged NS1 proteins with variations at position 95, 135, or 145, as well as V5-tagged NS1 proteins from human strains A/Brisbane/59/2007 (H1N1), A/Brisbane/10/2007 (H3N2), and A/Boston/DOA2-172/2013 (H3N2), were also unable to bind p85α (Fig. 6C and D). These data indicate that mutations that have accumulated in the p85β-binding site of NS1 proteins from human IAVs do not impact the selectivity of NS1 for p85β over p85α.

### Impact of NS1 p85β-binding variants on IAV replication and competitive fitness.

**FIG 7:**
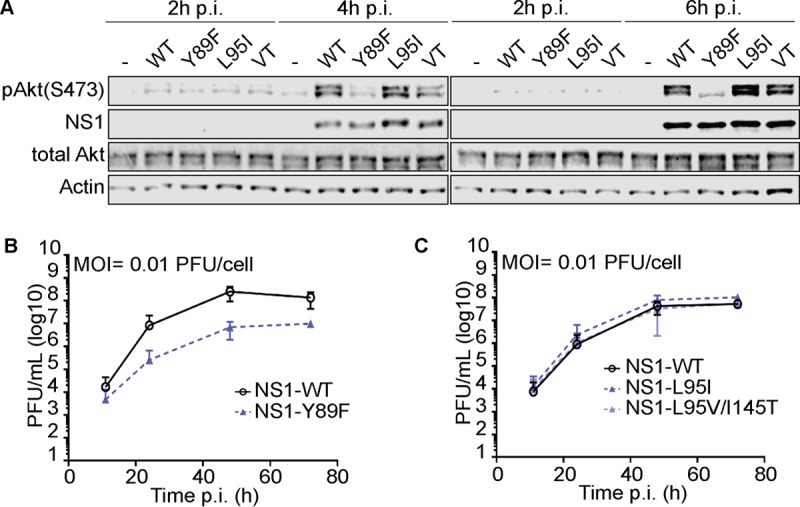
Impact of NS1 p85β-binding variants on IAV-induced Akt phosphorylation and replication *in vitro*. (A) Western blot analysis of total lysates of HAP1 cells infected with the indicated rPR8-based NS1 wild-type (WT) or mutant virus at an MOI of 5 PFU/cell for 2, 4, or 6 h. Proteins of interest were detected using specific antibodies. Data are representative of those from at least three independent experiments. VT, L95V/I145T. (B and C) Growth curve analysis of the indicated rPR8-based NS1 wild-type or mutant virus in A549 cells (MOI = 0.01 PFU/cell). Supernatants from the indicated time points were titrated in MDCK cells. Data points show mean values from three independent experiments ± SDs.

**FIG 8:**
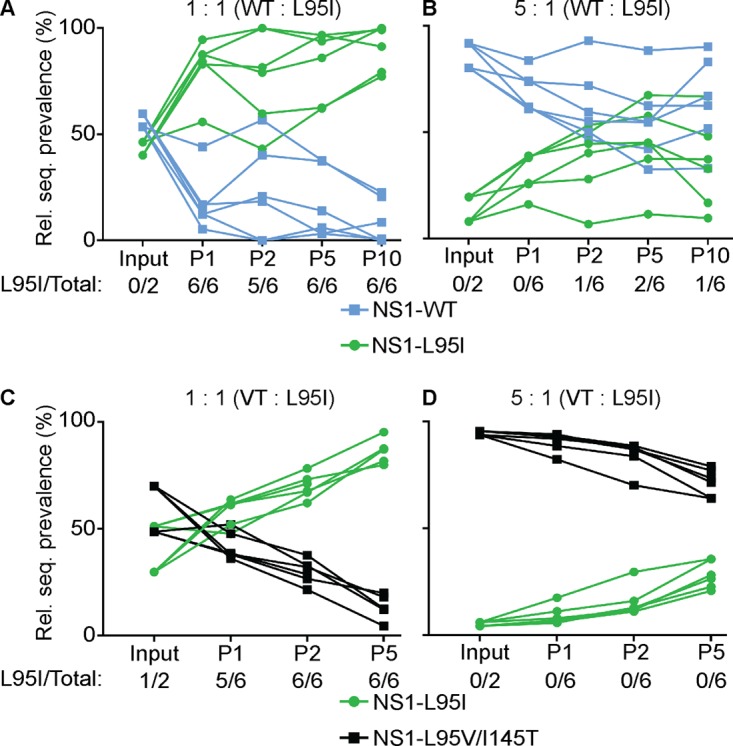
Impact of NS1 p85β-binding variants on IAV fitness *in vitro*. A549 cells were initially infected with the indicated mixture of rPR8-based NS1 wild-type (WT) or mutant virus at an MOI of 0.01 PFU/cell. For each passage, supernatants were harvested after 48 h, titrated, and used for subsequent passages at the same MOI. The graphs depict the relative prevalence of the indicated segment at each passage (P), as determined by Sanger sequencing and analysis of the relevant codon. Each point represents data from a single infected well of a single experiment (3 wells in each of 2 independent experiments). Rel. seq., relative sequence; VT, L95V/I145T.

The accumulation and maintenance of specific variants at the p85β-binding site in the NS1 proteins of circulating human IAVs may suggest that those variants provided a selective advantage to IAVs in humans. In order to test this hypothesis, we generated isogenic PR8-based viruses expressing NS1 proteins with the L95I amino acid change (PR8/NS1-L95I) or double L95V/I145T amino acid changes (PR8/NS1-L95V/I145T) to represent the p85β-binding capacity of H1N1 viruses isolated in about the 1940s or the late 2000s, respectively. In agreement with their impact on p85β binding, we found that these variants had predictable effects on activation of PI3K signaling during infection, as determined by Western blotting for the activated form of the downstream PI3K effector kinase, Akt: kinetic studies revealed a slightly enhanced level of phosphorylated Akt (pAkt) at later time points during infection with PR8/NS1-L95I compared to that during infection with the wild type, while PR8/NS1-L95V/I145T induced lower levels of pAkt at early time points (Fig. 7A). However, it was clear that both mutant viruses ultimately induced pAkt levels to a much greater extent than the PR8/NS1-Y89F control, whose NS1 protein lacks a detectable p85β-binding capacity. PR8/NS1-Y89F is attenuated for virus replication compared to wild-type virus in human lung epithelial A549 cells (Fig. 7B) (40). We therefore also assessed the replication kinetics of the PR8/NS1-L95I and PR8/NS1-L95V/I145T viruses to see if this correlated with their p85β-binding ability and level of pAkt induction. Perhaps reflecting the subtlety of these variants on the NS1-p85β interaction, we did not find a statistically significant enhancement of PR8/NS1-L95I virus replication or a defect in PR8/NS1-L95V/I145T virus replication compared to wild-type virus replication when measuring viral titers in the supernatants of infected A549 cells by a plaque assay (Fig. 7C). Nevertheless, we consistently observed that the PR8/NS1-L95I virus appeared to replicate slightly better than the wild type, although the titers were only ∼2-fold higher. To investigate this further, we therefore established a more sensitive competition-based assay in A549 cells where we could serially passage defined mixtures of the wild-type PR8 and PR8/NS1-L95I viruses to determine whether one could outcompete the other. Strikingly, at an input mixture ratio of 1:1 and in 6/6 replicates over 2 independent experiments, the PR8/NS1-L95I virus dominated over the wild-type virus within 1 to 2 passages (Fig. 8A). When a more stringent mixture ratio of 5:1 (wild type/mutant) was used, the PR8/NS1-L95I virus also showed a trend to outcompete the wild-type virus, although, as expected, this took more passages (Fig. 8B). Similarly, the PR8/NS1-L95I virus was able to outcompete the PR8/NS1-L95V/I145T virus (Fig. 8C and D). Parallel control passaging of each virus alone did not result in any spontaneous mutations in NS1. These data indicate that the naturally occurring 95I variant in the p85β-binding site of NS1 imposes a subtle growth advantage on a recombinant virus derived from a virus isolated in the 1930s. This experimental observation is consistent with the appearance of NS1 95I in human IAV strains in the 1940s, which effectively became fixed for the duration of the 20th century, and may suggest that this change was a contributor to effective adaptation to the human host.

### Covariation of NS1 p85β-binding phenotypes with functional changes in other IAV proteins.

While the observed reduced fitness of the PR8/NS1-L95V/I145T virus complements the findings from studies introducing artificial mutations into NS1 to destabilize its interaction with p85β (10, 40), it is surprising that a virus with such a phenotype of weakened binding presumably exhibited enhanced fitness in the human population to become dominant. We therefore speculate that other viral factors which were not effectively modeled in our tissue culture experiments using a noncontemporaneous recombinant virus may have contributed to the emergence of this phenotype *in vivo*. As a first step to uncover such factors, we performed evolutionary analyses on all human H1N1 and H3N2 viral proteins from viruses isolated over the last century to identify potential residues that covaried with H1N1 and H3N2 NS1 145T and 135N, respectively (summarized in Table 1). As expected, multiple amino acid variants occurred at these times during evolution, and functionally equivalent changes between H1N1 and H3N2 viruses (perhaps resulting from disparate amino acid changes) are difficult to determine. Nevertheless, we observed that in two viral proteins, NA and PB2, the same amino acid positions appeared to covary with the NS1 p85β-binding phenotype, even though such a phenotype arose independently in H1N1 and H3N2 viruses at least 15 years apart and arose with different causative sequence changes. In NA, S367 was replaced by either phenylalanine (H1N1) or asparagine (H3N2). Changes at this position have been functionally implicated in altering the second sialic acid-binding site in NA and are associated with avian-to-human host adaptation and, possibly, tissue tropism (48, 49). In PB2, T613 was replaced by either isoleucine (H1N1) or alanine (H3N2), and variants at this position have also been identified bioinformatically to be potential host signatures (50, 51). These preliminary analyses therefore identify at least two functional changes in NA and PB2 that covary with the NS1 p85β-binding phenotype during virus evolution in humans. This may provide a starting point to investigate the natural emergence of NS1 p85β-binding variants with apparently compromised fitness profiles *in vitro*.

**TABLE 1:** Human IAV residues varying with altered NS1 p85β-binding phenotypes

Virus, amino acid change	Residue(s) in IAV protein^*a*^:									
	HA	NA	PB1	PB2	PA	NP	M1	M2	NS1	NEP
H1N1										
NS1 L95I (enhanced p85β binding)	L78I, D85E, P89S, N142K, K147R, K177N, S202I, N211K, D239G, N293D, R497K	T86A, N200D, A232V, N270D, T332K, H369Q, R386N, S388F, R390K	N175D, M195V, L298I	A44S, I292T, A613T	S225C, A404S, S421I, I690V	R98K, R305K	I15V T218A	I28V, N31S R54L, Y57H, G89S, S93N	D53, D171N, E196K, G224R, stop231R	
NS1 I145T (reduced p85β binding)	K91E, K156N, N224S	P248A, I263V, S366R, S367F, N386D, L388S, I427V	R211K, R386K	R62K, V109I, K355N, V547I, T613I	S277F, A287S, Y321S, N350S	M105V, T373A		L36V, L43I, L54I	R231stop	I89T
H3N2, NS1 S135N (reduced p85β binding)	Q49R, S61N, T64I, K174N, K189Q, N205K, A214S, T228A, V239I, N294K, N328S, I377R, R466K	L81P, D93G, D147N, I215V, S367N, K369T, N387K, N402D, I464L	A587T	K353R, T613A	N409S	A127S	V219I		E26K, D139G, K229E	

a Underlined residues indicate positions that may covary with the p85β-binding phenotype, irrespective of subtype or NS1 variant.

## DISCUSSION

Although the binding of the IAV NS1 protein to host p85β and the subsequent activation of PI3K signaling are well documented (10–13, 39, 46), the downstream impact of this virus-host interaction has often been controversial (11, 41, 45), possibly because investigators use different virus strains, cell models, chemical inhibitors, or arbitrary NS1 mutants. In the study described here, we used a rational structure-guided approach to identify IAV NS1 mutants exhibiting a loss-of-function phenotype with respect to host p85β binding. Our results expand on the knowledge that Y89 is an essential NS1 determinant of this interaction by identifying 9 other NS1 residues that play additional roles, with the most important ones being residues 95, 98, 133, 145, and 162. These data provide a new panel of functionally defined mutants that can be used in future studies to elucidate the biological consequences of NS1 activation of PI3K signaling during IAV infection. This resource will allow a comparative phenotypic assessment of different viral mutants with a common functional defect, an experimental strategy that will limit the overinterpretation of data due to reliance on a mutant with a single arbitrary NS1 mutation. Given the multifunctionality of NS1 (1, 3), it is very likely that particular loss-of-function mutations can have more than one consequence. For example, it is known that some mutations used to disrupt the NS1-CPSF30 interaction also impact the ability of the NS1 effector domain to form homotypic interactions (52–54) and can have CPSF30-independent consequences (55). Therefore, biological studies performed with multiple independent NS1 mutants with common host protein binding properties are highly desirable to dissect NS1 functions. Our data can also be used to reinterpret the results of existing observational studies. For example, experiments aimed at adapting a human IAV isolate to mice identified a single NS1 variant at position 98 that increased virulence (98S) (38, 56). The molecular mechanisms underlying this phenotype might now also be considered in the context of the resource presented here that NS1 98S may exhibit altered p85β-binding properties.

We also used our rational structure-guided approach to assess the impact of naturally occurring amino acid variants in NS1 at its interface with p85β. This revealed that IAV NS1 proteins can exhibit a spectrum of p85β binding which is largely determined by variations at positions 95, 135, 145, 161, and 164. Strikingly, we observed that variants at positions 95, 135, and 145 arose and were maintained in human IAVs for periods of time over the last 100 years. This led to predictable alterations in the p85β-binding capacity: human IAV NS1 proteins with 95I (prevalent from the 1940s onwards) exhibited enhanced binding toward p85β, while independent variants at positions 135 and 145 destabilized p85β binding and became more prevalent in both H1N1 and H3N2 IAV NS1 proteins from the end of the 20th century onwards. In our experimental model system utilizing isogenic PR8-based viruses (isolated in 1934), we were able to demonstrate a competitive growth advantage for an IAV expressing NS1 with the 95I (p85β-binding-enhancing) variant, which would appear to be in line with its rapid selection in humans. This correlation suggests a benefit conferred to these viruses by enhanced p85β binding. Nevertheless, variants that destabilize p85β binding (135N or 145T) have recently emerged and been selected for in human IAVs, and our sensitive competition assay would suggest that, at least in the PR8-based background, such destabilizing variants are deleterious to virus propagation compared with the propagation ability of the preceding virus, which exhibited enhanced p85β binding (95I). How viruses expressing such NS1 variants therefore established themselves in the human population is unknown, and we can only speculate that compensatory or synergistic functional changes in other viral properties (highlighted in our evolutionary analyses as a potential interplay with NA or PB2 proteins) could have played a role.

It is currently unclear whether the evolution of the NS1 p85β-binding site in humans is directly related to a requirement to alter a PI3K-related function. Indeed, it also remains to be determined whether the subtle differences in fitness phenotypes that we observed are dependent upon p85β. Our assays revealed that even IAV NS1 proteins with partially weakened p85β binding were able to stimulate PI3K activity to a substantial extent at later times of infection. Thus, it may be that the natural magnitude of IAV NS1-induced PI3K activation can vary if the net biological consequences (i.e., downstream signaling) remain unaffected. In this regard, it is not inconceivable that the p85β-binding capacity is maintained within a flexible range but is in conflict with variant selection that promotes NS1 functional adaptation to other host factors, for example, TRIM25 or Riplet, to which NS1 binding is species specific and involves NS1 residues close to the p85β-binding site (4). Further studies are required to investigate such possibilities, as well as the potential interdependence of p85β-binding evolution with other viral factors.

In summary, the data resource presented here can be used for the rapid, sequence-based prediction of the p85β-binding capacity of any given IAV NS1 protein. Although potential correlations with the host range, replicative capacity, or virulence must still be determined using additional sensitive experimental models, this information will be invaluable for fully characterizing the biology of both newly emerging IAVs in the future and IAVs of interest as they evolve in particular hosts.

## MATERIALS AND METHODS

### Cells.

MDCK, 293T, and A549 cells were cultured in Dulbecco's modified Eagle medium (DMEM) supplemented with 10% fetal bovine serum (FBS), 100 units/ml penicillin, and 100 μg/ml streptomycin (Gibco Life Technologies). HAP1 cells (Horizon Discovery, Vienna, Austria) were cultured in Iscove's modified Dulbecco's medium (IMDM) supplemented with the same additives mentioned above as well as 200 μM GlutaMAX (Gibco Life Technologies). All cells were maintained at 37°C with 5% CO_2_.

### Construction of plasmids.

The NS1 cDNA sequences from A/Puerto Rico/8/1934 (PR8; H1N1), A/California/04/09 (Cal09; 2009 pandemic H1N1), A/Brisbane/59/2007 (H1N1), A/Brisbane/10/2007 (H3N2), and A/Boston/DOA2-172/2013 (H3N2) were PCR amplified from existing plasmids (or reverse transcription-PCR [RT-PCR] products) and were ligated in frame with an N-terminal V5 tag in a modified pLVX-IRES-ZsGreen1 plasmid (Clontech, CA). A pLVX-IRES-ZsGreen1 plasmid carrying GST with a C-terminal V5 tag was generated in a similar manner. A two-step overlap PCR was used to introduce site-directed mutations into the cDNAs as required. All NS1 cDNAs also contained silent mutations to prevent expression of the nuclear export protein (NEP) and were modified as required to prevent NS1 binding to CPSF30 (103S/106I). The pPolI-based construct expressing the wild-type PR8 NS segment was kindly provided by Adolfo García-Sastre (Icahn School of Medicine at Mount Sinai, New York, NY, USA) and was used as a template for site-directed mutagenesis reactions using a QuikChange II site-directed mutagenesis kit (Stratagene, TX). The identity of all constructs used was confirmed by sequencing.

### Coimmunoprecipitation analysis.

Using the FuGENE 6 reagent (Promega), 293T cells seeded in 25-cm^2^ flasks were transfected with 2 μg of the pLVX-IRES-ZsGreen1 plasmid (Clontech, CA) carrying V5-tagged NS1 proteins or GST (as appropriate) and 1 μg of the p3×FLAG-CMV7.1 plasmid (Sigma-Aldrich, MO) expressing FLAG-tagged human p85β or human p85α (provided by Hannah L. Turkington, University of Zurich, Zurich, Switzerland). At 48 h posttransfection, cells were lysed in 1 ml of passive lysis buffer (50 mM Tris-HCl [pH 7.8], 500 mM NaCl, 5 mM EDTA, 0.5% Nonidet P-40 substitute) supplemented with a cOmplete Mini protease inhibitor tablet as indicated by the manufacturer (Roche). Following sonication and clarification by centrifugation, soluble fractions were incubated for 2 h at 4°C with anti-V5 antibody (Bio-Rad). Lysate-antibody mixes were then incubated overnight at 4°C with protein G-Sepharose beads (Sigma-Aldrich). Following extensive washing with passive lysis buffer, a urea-based disruption buffer (6 M urea, 2 M β-mercaptoethanol, 4% SDS) was used to dissociate precipitated proteins from the beads. Samples were stored at −20°C until analysis by SDS-PAGE and Western blotting.

### Construction and functional analysis of recombinant viruses.

Recombinant A/Puerto Rico/8/1934 (rPR8) viruses were rescued and titrated essentially as described previously (57). Briefly, using the FuGENE 6 reagent (Promega), 6 × 10^5^ 293T cells were seeded in 6-well plates and cotransfected 24 h later with 7 ambisense pDZ-based expression plasmids expressing the PR8 segments nucleoprotein (NP), PA, PB1, PB2, matrix (M), hemagglutinin (HA), and neuraminidase (NA) and a pPolI-based plasmid carrying the PR8 NS segment. At 24 h posttransfection, the cells were washed once in sterile phosphate-buffered saline (PBS) and 3 × 10^5^ MDCK cells in DMEM supplemented with 1 μg/ml tosylsulfonyl phenylalanyl chloromethyl ketone (TPCK)-treated trypsin (Sigma-Aldrich, MO) were added. Forty-eight hours later, the supernatants were harvested, the viruses were plaque purified, and virus stocks were grown and titrated using standard methods in MDCK cells. RNA was extracted from stock aliquots using a ReliaPrep RNA tissue miniprep system (Promega), and the NS genomic segments of each virus were fully sequenced after segment-specific RT-PCR to ensure the absence of undesired mutations.

To determine virus replication kinetics, 5 × 10^5^ A549 cells were seeded in 12-well plates and infected 24 h later with the indicated virus diluted in PBS supplemented with 100 units/ml penicillin and 100 μg/ml streptomycin (Gibco Life Technologies), 0.45% bovine serum albumin (Sigma-Aldrich), and 1 mM Ca^2+^-Mg^2+^ at a multiplicity of infection (MOI) of 0.01 PFU/cell. Following 1 h of adsorption, cells were washed three times in FBS-free DMEM and incubated in FBS-free DMEM supplemented with 0.5 μg/ml TPCK-treated trypsin (Sigma-Aldrich, MO). The supernatants were harvested at the times indicated above and titrated by plaque assay on MDCK cells.

To perform competition assays, 5 × 10^5^ A549 cells were seeded in 12-well plates and infected 16 to 24 h later with the mixed inocula (ratios of 1:1 or 5:1) indicated above at an MOI of 0.01 PFU/cell. Following 1 h of adsorption, the cells were treated as described above. The supernatants were collected at 48 h postinfection (p.i.) (passage 1), titrated by plaque assay on MDCK cells, and used for repeated infection of A549 cells to generate virus for passage 2. This procedure was repeated until the passage number indicated above. RNA was extracted from the supernatants of each passage and the input inoculum using a ReliaPrep RNA tissue miniprep system (Promega), and the NS genomic segments were fully sequenced after segment-specific RT-PCR. Quantification of NS segment prevalence was performed by determining the relative peak height of the unique bases of mutant or wild-type codons using BioEdit sequence alignment editor software following commercial Sanger sequencing (GATC Biotech, Germany).

To determine the levels of virus-induced Akt phosphorylation, 5 × 10^5^ HAP1 cells were seeded in poly-l-lysine (Sigma-Aldrich)-precoated 12-well plates. Following attachment, the cells were washed three times prior to incubation overnight in serum-free IMDM. The cells were infected with the virus indicated above diluted in PBS supplemented with 100 units/ml penicillin and 100 μg/ml streptomycin (Gibco Life Technologies), 0.45% bovine serum albumin (Sigma-Aldrich), and 1 mM Ca^2+^-Mg^2+^ for 1 h at an MOI of 5 PFU/cell. Following three washes with PBS, infected cells were maintained in FBS-free IMDM, and total protein lysates were harvested at the times postinfection indicated above in a urea-based disruption buffer (6 M urea, 2 M β-mercaptoethanol, 4% SDS). Samples were stored at −20°C until analysis by SDS-PAGE and Western blotting.

### SDS-PAGE and quantitative Western blotting.

Samples were sonicated to shear the nucleic acids and then boiled for 5 min. Polypeptides were resolved by SDS-PAGE on NuPAGE 4 to 12% bis-Tris protein gels (Thermo Fisher), followed by transfer to nitrocellulose membranes. Proteins were detected by Western blotting using the following primary antibodies: mouse anti-V5 (catalog number MCA1360; Bio-Rad), mouse anti-FLAG (catalog number F1804; Sigma-Aldrich), rabbit anti-actin (catalog number A2103; Sigma-Aldrich), mouse anti-Akt (catalog number 2920; Cell Signaling Technology), and rabbit anti-pAkt (phosphorylated at S473; catalog number 4060; Cell Signaling Technology). Secondary antibodies were fluorochrome-conjugated anti-mouse immunoglobulin (catalog number 35519; Thermo Fisher Scientific) and anti-rabbit immunoglobulin (catalog number SA5-10036; Thermo Fisher Scientific,). A Li-Cor Odyssey scanner was used for detection.

### Structural and bioinformatic analyses.

Structural representations were visualized using the appropriate Protein Data Bank (PDB) file and the PyMOL program (58). All NS1 protein sequence data were obtained and analyzed using tools from the NIAID Influenza Research Database (IRD) (47) (http://www.fludb.org).

### Evolutionary analyses.

IAV sequences (almost complete open reading frames, preferably from isolates for which all eight segments have been sequenced) were downloaded from the NCBI Influenza Virus Database (59) and aligned using the MEGA (version 5) program (60). A preliminary phylogenetic tree of each segment for which >500 sequences were available was calculated, and most of the redundant sequences were removed to yield alignments containing between 100 and 200 representative sequences. The alignments were used for tree inference with the MEGA (version 5) program (neighbor-joining method; substitution model, uncorrected p distance; units, number of amino acid differences per site). Statistical support was estimated with a bootstrap test (1,000 replicates). Persisting amino acid exchanges correlating with the L95I, I145T, and S135N substitutions of NS1 were identified in the alignments.
